# The role of morphological adaptability in *Vibrio cholerae*’s motility

**DOI:** 10.1128/mbio.02469-24

**Published:** 2024-11-29

**Authors:** Jun Xu, Keigo Abe, Toshio Kodama, Marzia Sultana, Denise Chac, Susan M. Markiewicz, Hideyuki Matsunami, Erika Kuba, Shiyu Tsunoda, Munirul Alam, Ana A. Weil, Shuichi Nakamura, Tetsu Yamashiro

**Affiliations:** 1 Department of Bacteriology, Graduate School of Medicine, University of the Ryukyus, Nishihara, Okinawa, Japan; 2 Department of Applied Physics, Graduate School of Engineering, Tohoku University, Sendai, Miyagi, Japan; 3 Department of Bacteriology, Institute of Tropical Medicine, Nagasaki University, Nagasaki, Japan; 4 Infectious Diseases Division, International Center for Diarrheal Disease Research, Bangladesh, Bangladesh, Dhaka; 5 Department of Medicine, University of Washington, Seattle, Washington, USA; 6 Molecular Cryo-Electron Microscopy Unit, Okinawa Institute of Science and Technology, Okinawa, Japan; University of South Florida, Tampa, Florida, USA

**Keywords:** *Vibrio cholerae*, bacterial filamentation, bacterial motility, mucus penetration

## Abstract

*Vibrio cholerae,* the causative agent of cholera, displays remarkable adaptability to diverse environmental conditions through morphological changes that enhance its pathogenicity and influence the global epidemiology of the disease. This study examines the motility differences between filamentous and comma-shaped forms of the *V. cholerae* O1 strain under various viscosity conditions. Utilizing the El Tor strain, we induced filamentous transformation and conducted a comparative analysis with the canonical comma-shaped morphology. Our methodology involved assessing motility patterns, swimming speeds, rotation rates, kinematics, and reversal frequencies using dark-field microscopy and high-speed imaging techniques. The results show that filamentous *V. cholerae* cells retain enhanced motility in viscous environments, indicating an evolutionary adaptation for survival in varied habitats, particularly the human gastrointestinal tract. Filamentous forms exhibited increased reversal behavior at mucin interfaces, suggesting an advantage in penetrating the mucus layer. Furthermore, the presence of filamentous cells in bile-supplemented medium underscores their relevance in natural infection scenarios.

**IMPORTANCE:**

This study highlights the enhanced motility of filamentous *Vibrio cholerae* in viscous environments, an adaptation that may provide a survival advantage in the human gastrointestinal tract. By demonstrating increased reversal behavior at mucin interfaces, filamentous *V. cholerae* cells exhibit a superior ability to penetrate the mucus layer, which is crucial for effective colonization and infection. Filamentous cells in bile-supplemented media further underscores their potential role in disease pathogenesis. These findings offer critical insights into the morphological flexibility of *V. cholerae* and its potential implications for infection dynamics, paving the way for more effective strategies in managing and preventing cholera outbreaks.

## INTRODUCTION

*Vibrio cholerae*, a Gram-negative, curved rod, comma-shaped bacterium, the causative agent of cholera, is a significant global public health concern, particularly in regions with limited access to clean water and sanitation (1). *V. cholerae* is responsible for severe diarrheal outbreaks, leading to high morbidity and mortality rates worldwide. The ongoing challenge of cholera, characterized by millions of cases annually, predominantly in under-resourced areas, underscores the critical need for a comprehensive understanding of this pathogen’s biology and mechanisms of disease (2). The impact of cholera extends beyond immediate health implications, affecting socio-economic stability and development. Rapid spread in communities, potential for severe dehydration, and high fatality rates in untreated cases make cholera a key target for public health interventions (3). The disease often highlights broader issues related to water safety and public health infrastructure, serving as an indicator of environmental and societal health (4).

At the molecular level, the virulence of *V. cholerae* is primarily attributed to the cholera toxin, which disrupts intestinal electrolyte balance, causing the characteristic watery diarrheal disease (5). In addition, bacterial motility, facilitated by a single polar flagellum, plays a critical role in its ability to colonize the human intestine and navigate through aquatic environments (6). This motility is multifaceted, contributing not just to locomotion but also to biofilm formation, environmental sensing, and host-tissue interactions (7–10). The motility of *V. cholerae* is a complex and finely regulated process, involving a series of cellular and molecular mechanisms that enable the bacterium to respond to environmental cues and adapt to varying conditions.

Despite extensive research, *V. cholerae* continues to reveal complexities in its adaptation strategies. Under specific environmental conditions, it can undergo a dramatic morphological transformation into an elongated, helical filamentous form (11). This change raises important questions about its impact on bacterial motility and pathogenicity. The filamentous form of *V. cholerae* represents a significant deviation from its typical morphology, potentially altering its swimming behavior, colonization efficiency, and interactions with host tissues (12, 13). Motivated by a previous study indicating that filamentation in *V. cholerae* enhances its attachment to chitin surfaces at the expense of competition within biofilms (13), we sought to further investigate the motility characteristics of filamentous cells. Understanding the motility of filamentous *V. cholerae* may reveal additional adaptive advantages in various environmental contexts, including the human gastrointestinal tract.

## RESULTS

### Filamentation of *V. cholerae* cells

**Fig 1:**
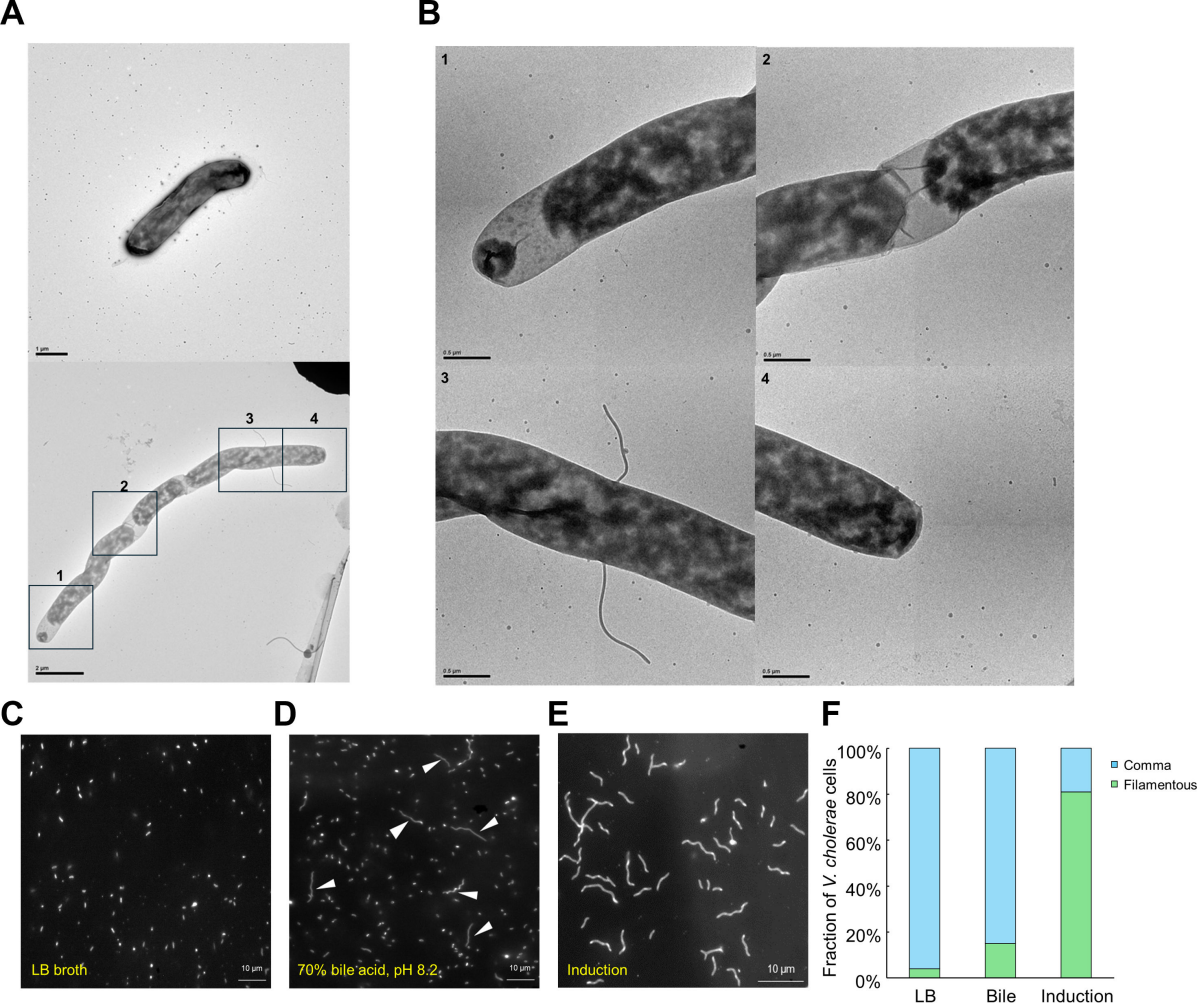
(**A**) Transmission electron microscopic images of comma-shaped (upper) and filamentous (lower) *V. cholerae* cells. (**B**) Enlarged transmission electron microscopic images detailing the filamentous cell body. (**C**) Dark-field microscopic image illustrating typical *V. cholerae* cells in Luria-Bertani (LB) medium. (**D**) Dark-field microscopic image showing the filamentation of *V. cholerae* cells cultured in LB medium supplemented with bile. White arrows indicate filamentous cells. (**E**) Dark-field microscopic image of *V. cholerae* cells after 18 hours of induction. (**F**) Fraction of *V. cholerae* cells that transformed into filamentous forms in LB medium, bile-supplemented LB medium, and the induction solution, comparing conditions over an 18-hour period. Data are aggregated from a sample size of *n* = 150 cells for each condition, compiled from three independent experimental trials.

*Vibrio cholerae* can alter its cell morphology from comma-shaped to filamentous in response to various environmental cues, such as insufficient nutrient supply and chemical stress from antibiotic treatment, often as a result of unsuccessful binary fission (13, 14). Filamentous cells typically measure around 10 µm in length, which is approximately 10 times the average length of comma-shaped cells (Fig. 1A and B; Table S2). When cultured in L-broth supplemented with 70% bile, approximately 15% of *V. cholerae* cells exhibited filamentation within a few hours (Fig. 1D and F). Both cell types maintained swimming motility, with comma-shaped cells swimming at approximately 35 µm/s and filamentous cells at about 10 µm/s (Fig. S1). This observation suggests the possible natural formation of filamentous *V. cholerae* within the human small intestine implies a potential role for filamentous *V. cholerae* O1 in the pathogenesis of cholera.

To better study the motility and other characteristics of filamentous cells, it was necessary to increase the proportion of filamentous cells in the culture. Therefore, we developed an induction method using antibiotics modified from previous methodologies (14, 15). We induced the transformation of comma-shaped *V. cholerae* cells into their filamentous form using ampicillin (Fig. 1C and E). Notably, this morphological change did not lead to a loss of motility. Instead, the filamentous cells retained their ability to move, although their motility characteristics, including speed and movement mechanisms, were modified to reflect their altered morphology. Our induction method successfully transformed over 80% of the cells into filamentous form within 3 hours (Fig. 1F; Movie S1).

A time-course observation confirmed the stability of filamentation, cell growth, and morphology *via* microscopy (Fig. S2). The results showed that filamentation remained stable under the inducing conditions. Although *V. cholerae* cells exhibited motility and filamentation, the minimal amount of nutrients in the induction solution limited the replication of cells (Fig. S2B). To further assess the physiological impact of filamentation, we measured ATP levels throughout the induction process, confirming that filamentation did not compromise cellular function (Fig. S2C).

The filamentous and motile states of *V. cholerae* were maintained as long as the cells remained in the inducing environment. Upon removal of the inducing agents or transfer to nutrient-rich conditions, the cells gradually reverted to their typical comma-shaped morphology. All experiments were conducted within the stable filamentation window (after 3 hours of induction), ensuring that the observed behaviors and characteristics reflect the filamentous state rather than transient physiological changes.

### Swimming pattern of filamentous *V. cholerae* cells

**Fig 2:**
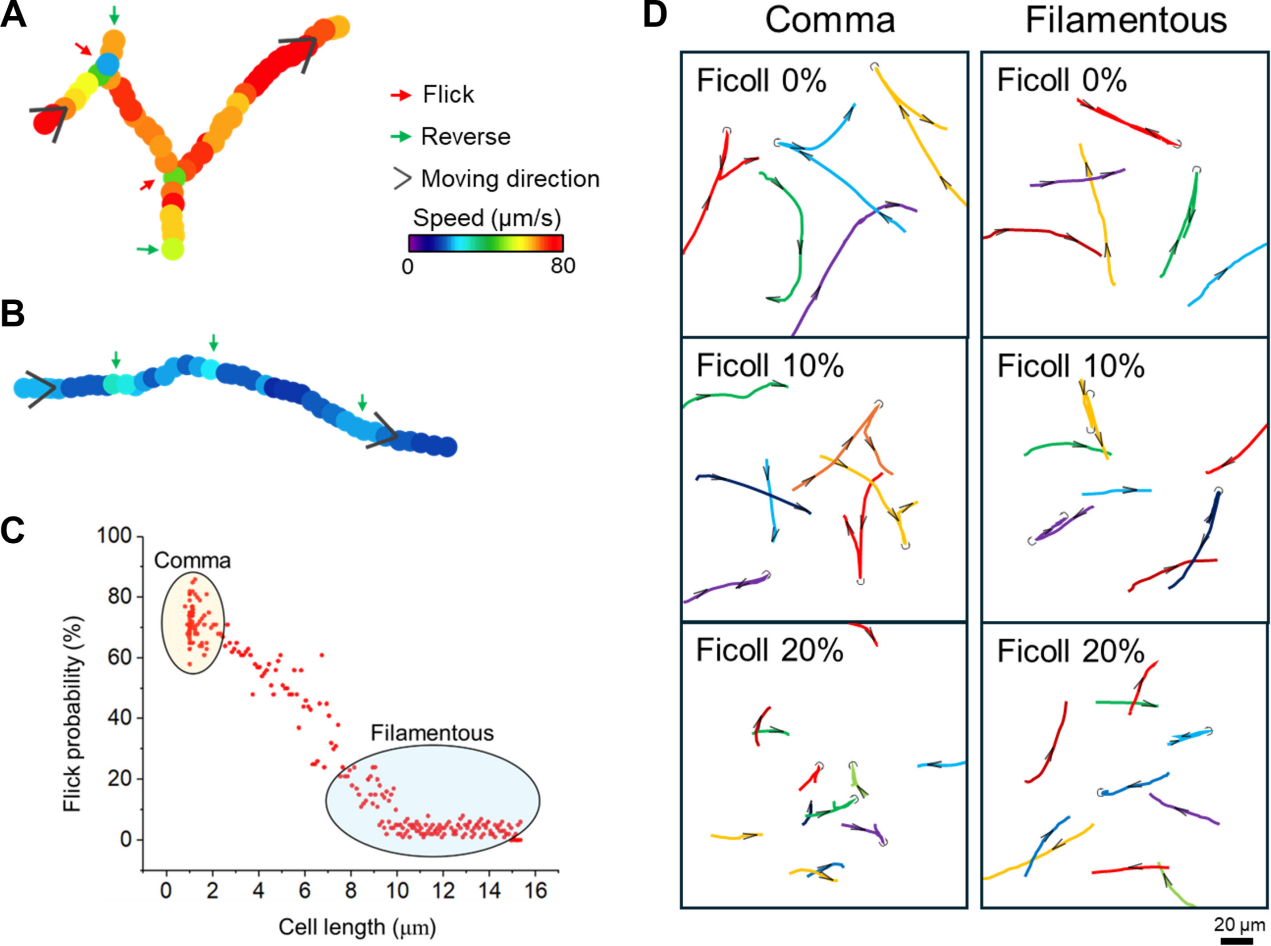
Motility pattern of *V. cholerae* cells. (**A**) and (**B**) illustrate the typical swimming pattern of a comma-shaped cell and a filamentous cell, respectively. Red arrows highlight instances of “flicking” movements—a rapid change in direction facilitated by the bacterium’s flagellum, with green arrows indicating moments of reversal in the cell’s trajectory. Black arrows represent the general direction of movement, while the variation in swimming speeds is conveyed through color coding, providing an intuitive visualization of speed differences. (**C**) Probability of flicking during a swimming cycle (forward-reverse-backward-reverse-forward) versus cell length in low-viscosity medium. Cells that flicked after forwarding onset were scored 100% probability, whereas cells that did not flick were scored 0% probability. Data were collected from over 300 cells of each form. (**D**) displays the swimming trajectories over a 5-second period for representative *V. cholerae* cells in environments of varying viscosities. Black arrows and U-turn symbols illustrate the direction of movement and instances of reversal maneuvers, respectively.

Filamentous cells showed distinct swimming patterns compared to the typical “shooting star” movement seen in comma-shaped cells (Movie S2). While they lost the ability to perform the “flick” movement (16) typical of the latter (Fig. 2A and C), they adapted to turning maneuvers with large radius or “back and forth” movements, particularly when changing direction (Fig. 2B and C). Figure 2D demonstrates how the swimming patterns of *V. cholerae* cells are influenced by changes in the viscosity of their environment. In high-viscosity environments, comma-shaped cells exhibited reduced travel distance (Fig. 2D, left graph), whereas filamentous cells’ movement patterns remained relatively less affected (Fig. 2D, right graph).

### Swimming kinematics of filamentous *V. cholerae* cells

**Fig 3:**
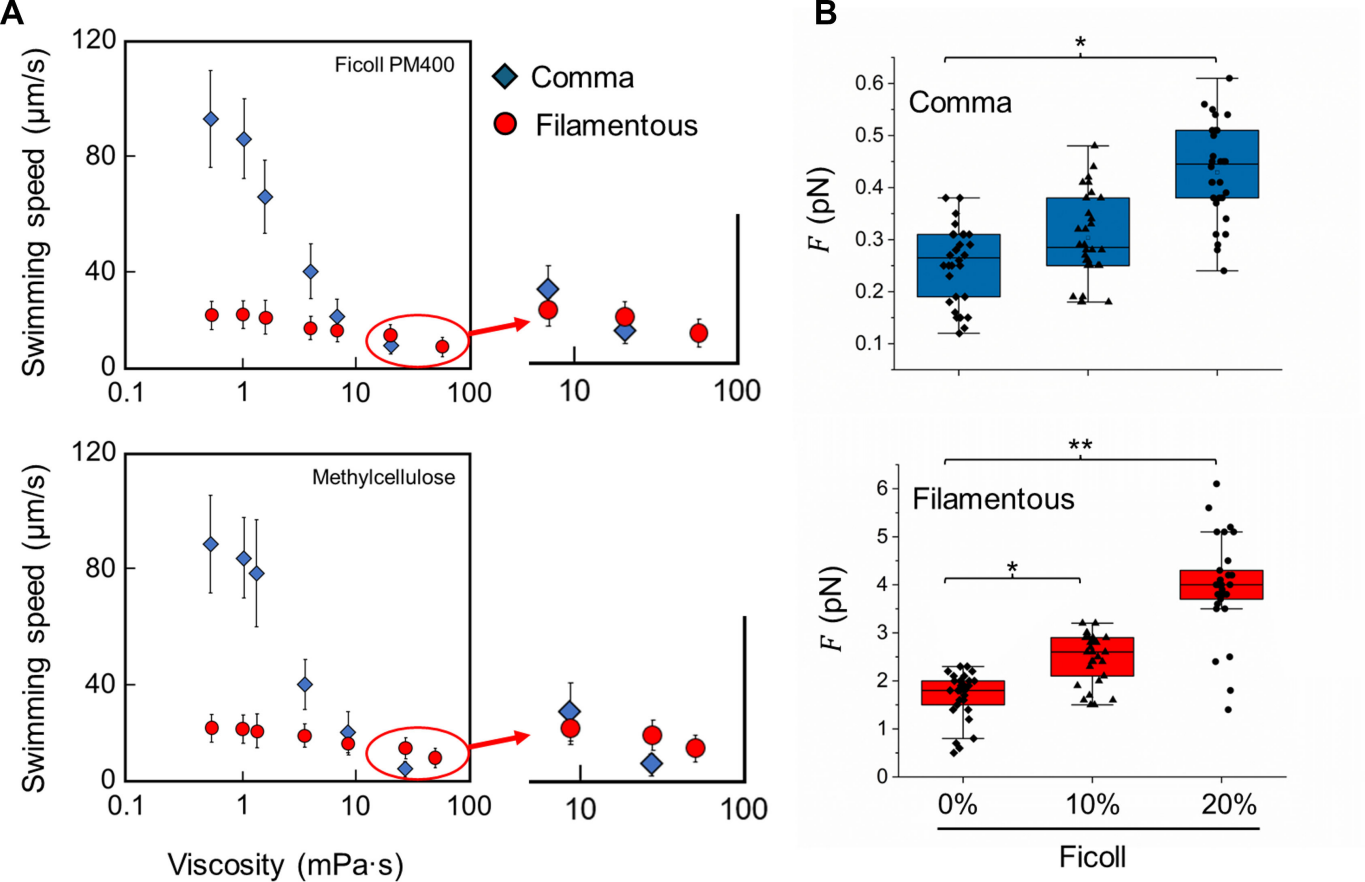
Kinematic parameters of *V. cholerae* cells. (**A**) presents the average swimming speeds of both comma-shaped and filamentous *V. cholerae* cells in media with varying viscosities. The enlarged insets provide a closer look at the bacterial motility in highly viscous environments. (**B**) The box charts detail the force exerted on the two morphological forms of *V. cholerae* while swimming in media with different viscosities. A one-way ANOVA with Dunnett’s test was conducted to assess the statistical significance of the observed differences in force exertion, with asterisks indicating significant differences (*P < 0.05, **P < 0.01). The data are aggregated from a sample size of *n* = 60 cells for each viscosity level, compiled from three independent experimental trials.

In low-viscosity media such as water, filamentous cells swim at an average speed of 20 µm/s, slower than the 100 µm/s average in comma-shaped cells (Fig. 3A). There is a clear correlation between increased body length and decreased swimming speed (Fig. S3, black dots/line). Both cell types displayed viscosity-dependent motility in both Newtonian (Ficoll) and non-Newtonian (Methylcellulose) fluids, with dramatic impairments in comma-shaped cells but less impact to filamentous cells at viscosities above 10 mPa•s (Fig. 3A), which is similar to the viscosity of the mucus layer in the small intestine (17, 18).

Filamentous cells exerted significantly higher forces during locomotion compared to comma-shaped cells. Specifically, the propulsive force for comma-shaped cells is approximately 0.2 pN in low-viscosity media, while filamentous cells generate nearly 10 times this force (Fig. 3B). Longer cell bodies tend to generate higher forces during locomotion (Fig. S3, red dots/line). This substantial difference in force generation highlights the powerful locomotion of the filamentous form and its adaptability to high-viscosity environments. Furthermore, filamentous cells generate about 2,000 pN nm of torque at a cell body rotation rate of 10 Hz in a motility buffer, with a linear decrease in torque as Ficoll concentration increases (Fig. S4). This level of torque is even comparable to that generated by large cells such as the spirochete *Leptospira* (19).

### Reversal behavior during swimming and penetrability of filamentous cell

**Fig 4:**
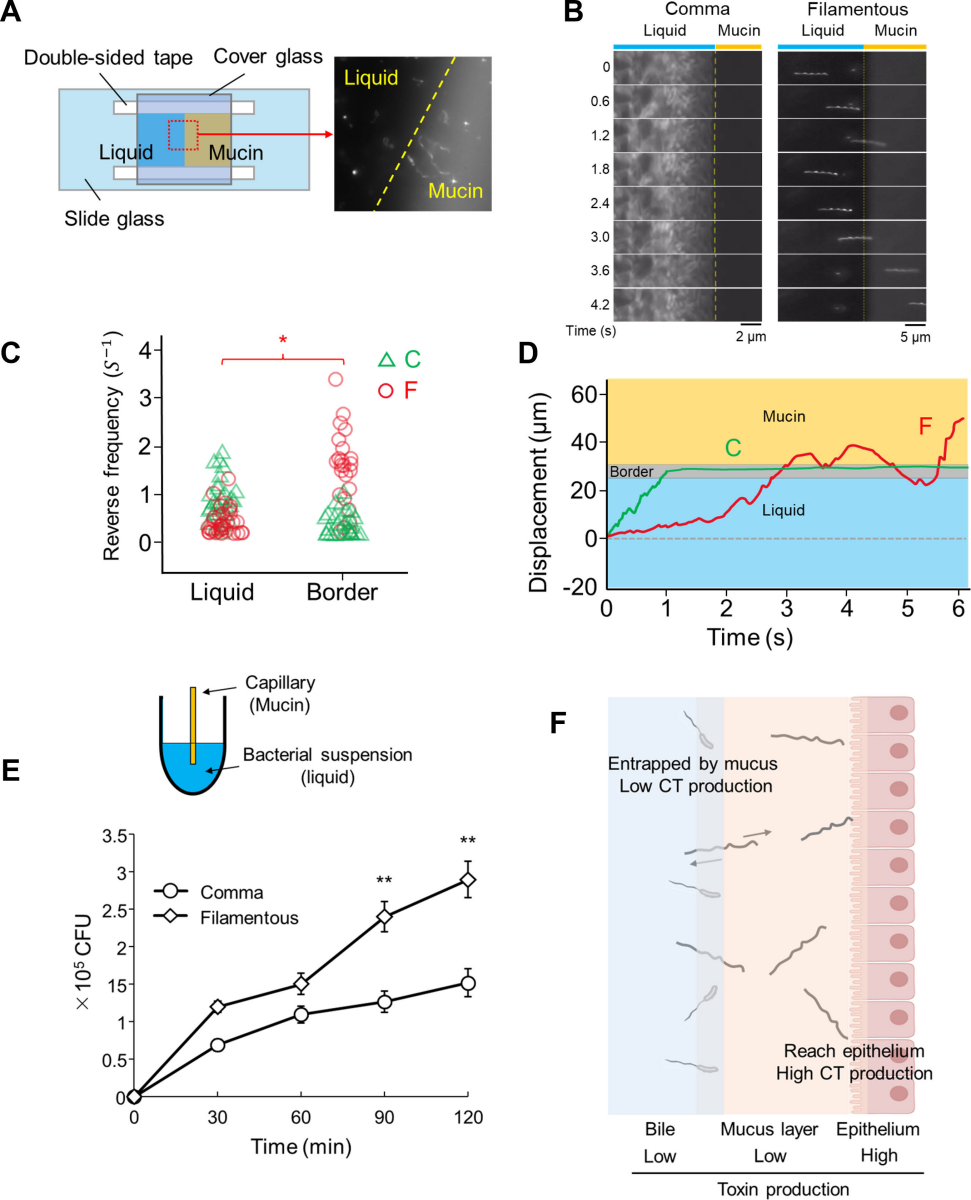
Penetrability of filamentous *V. cholerae* cells. (**A**) A schematic representation of the flow chamber setup containing adjoining liquid bile phase and mucin phase. The initial positioning of bacterial cells is in the liquid phase at the start of the experiment. (**B**) A graph depicting the reversal frequency of comma-shaped (green triangle) and filamentous (red circle) cells in both the liquid phase and at the border. A Mann-Whitney U test revealed a significant difference in the reversal frequencies of filamentous cells between the liquid phase and the border area (*P < 0.05), with *n* = 20 cells evaluated for each condition. (**C**) Time course of the locomotion of comma-shaped and filamentous cells observed at the liquid-mucin border. (**D**) Tracks illustrate the displacement of individual comma-shaped (indicated by “C”) and filamentous (indicated by “F”) cells as they swim in the liquid and at the border between the liquid and mucin phases. The gray band represents the border’s position. (**E**) Schematic of the capillary assay (upper) and cell number of *V. cholerae* in the capillary (lower) (**P < 0.01). (**F**) A schematic illustration of how frequent reversal movements in filamentous cells might contribute to their navigation.

To observe the detailed swimming behavior of *V. cholerae* cells, we utilized a flow chamber setup designed to mimic the natural aquatic environments of *V. cholerae*. The flow chamber consists of two main phases: a liquid bile phase and a mucin phase, which together create an interface that simulates the gradient encountered by *V. cholerae* at the boundary between aqueous environments and the more viscous mucus layers of the human intestine (Fig. 4A).

Filamentous *V. cholerae* cells exhibited a unique reversal behavior at the liquid-mucin interface (Fig. 4B), characterized by distinct “back-and-forth” movements (Fig. 4C and D; Movie S4). This behavior sharply contrasts with that of the more agile comma-shaped cells, which became entrapped at the same interface (Fig. 4C and D; Movie S3). The frequent and pronounced reversal movements of filamentous cells suggest an adaptive mechanism for efficient navigation in viscous environments. The “back-and-forth” motion demonstrated by these cells appears to be a strategy to explore and potentially penetrate the viscous mucus barrier, seeking more accessible routes through this physical obstacle. We further performed a capillary assay to quantify the effect of this motion. The result indicates that filamentous cells are more capable of translocating into the mucous zone by showing a higher cell number in it (Fig. 4E), consistent with the results of the flow chamber assay. This strategy in motion could be attributed to the much stronger force generated by the filamentous cells, potentially facilitating the penetration of mucus layers and allowing the cells to dive deeper—an essential component of the bacterium’s pathogenic process such as toxin production (Fig. 4F).

## DISCUSSION

*Vibrio cholerae* remains a formidable public health challenge, particularly in regions with inadequate water and sanitation infrastructure. The adaptability and resilience of *V. cholerae*, notably in its ability to assume different morphological forms, have long been subjects of interest in microbiological research (20, 21).

Our findings reveal that the filamentous form of *V. cholerae*, despite its reduced swimming speed, exhibits less-affected motility by the change of viscosity, suggesting an evolutionary adaptation for navigating and surviving in diverse habitats, including the human gastrointestinal tract (22, 23). When contaminated food or water is ingested, most *V. cholerae* cells are destroyed by stomach acid (24). However, a small fraction survives to reach the small intestine lumen, often in the form of biofilms or microcolonies (25). Here, bacterial cells navigate through bile and mucus layers to reach and adhere to epithelial cells (26), where they proliferate, produce toxins, and initiate infection (27).

Our study also shows that morphological transformation does not compromise *V. cholerae*’s toxin-producing ability. Both filamentous and comma-shaped forms exhibit comparable expression levels of the *ctxA* gene (Fig. S5), indicating their potential to induce cholera-like symptoms (10, 28). Efficient motility for reaching the epithelial surface of the small intestine is crucial, as most cholera toxin (CT) production occurs after *V. cholerae* cells adhere and colonize to epithelium due to transcriptional regulation (29–31). While comma-shaped cells exhibit high swimming speeds, they generate low force and are significantly affected by changes in viscosity. By contrast, filamentous cells demonstrate robust motility under high-viscosity conditions and frequent “back-and-forth” movements, enhancing their ability to traverse the mucus layer and efficiently reach the epithelial cells, where toxin production occurs (Fig. 4F). Although our liquid-mucin experiments may not fully replicate the rheological properties of intestinal mucin gels, the ability of filamentous cells to perform frequent reversal movements at mucin borders is noteworthy. This behavior likely facilitates mucus layer penetration, an essential factor in cholera pathogenesis (32, 33), providing an advantage over comma-shaped cells in reaching the intestinal epithelium.

Crucially, the presence of filamentous *V. cholerae* in the cultural medium with bile and potentially in the clinical watery stool samples from patients with cholera (Fig. S6) hinted the relevance of these morphological forms in natural infection scenarios, echoing findings from previous clinical studies (31). This correlation between laboratory and clinical observations underscores the significance of the filamentous form in the natural course of *V. cholerae* infections. The filamentous morphology could offer several advantages in terms of motility, particularly in navigating viscous environments such as mucus layers, which may facilitate more efficient colonization of the host’s intestinal tract. This enhanced motility increases the likelihood of the bacteria successfully reaching the epithelial cells and establishing infection. In addition, the physical robustness and increased surface area of filamentous cells may enhance adherence and better survival on various surfaces, including food and water sources, thereby increasing the opportunities for ingestion and infection of new hosts (12, 13). Although the direct contribution to pathogenesis requires further exploration, these advantages of motility suggest that filamentous cells can play a potential role in the persistence and transmission of *V. cholerae*.

On the other hand, the structural adaptations of the flagella during filamentation remain an important area for further investigation. Preliminary data suggest that filamentous cells possess shorter flagellum at the terminal segment of their elongated bodies, raising questions about how these structural modifications influence motility and torque generation. Understanding how flagellar adaptations contribute to the enhanced motility of filamentous cells in high-viscosity environments will be essential to providing a more comprehensive picture of *V. cholerae*’s survival strategies.

In summary, we analyzed the motility characteristics of filamentous and comma-shaped *V. cholerae* under varying environmental conditions. Our study demonstrated that filamentous *V. cholerae* cells exhibit enhanced motility in viscous environments compared to their comma-shaped counterparts. We observed that filamentous cells retained their motility and performed frequent reversal movements, facilitating their navigation through mucus layers. These findings suggest that filamentous *V. cholerae* cells may possess a distinct advantage in traversing the gastrointestinal environment, potentially aiding in their ability to reach the epithelial surface where cholera toxin production occurs. Our work underscores the importance of morphological transformation in *V. cholerae*’s survival and infection strategies, providing a foundation for further investigations into the mechanisms and implications of these morphological changes.

## MATERIALS AND METHODS

### Bacterial strain, culture medium, and reagents

The clinically isolated *V. cholerae* O1 El Tor strain N16961 (ATCC 39315), known for its well-characterized flagellar and chemotactic systems, was selected for this study to accurately assess changes in motility. Bacterial cultures were grown in Luria-Bertani (LB) broth at 37°C, maintaining a pH of 7.5. The LB medium comprised 10 g of tryptone, 5 g of yeast extract, and 5 g of NaCl per liter (Nacalai Tesque, Kyoto, Japan). For observing motility, *V. cholerae* cells were resuspended in TMN (Tris-MgCl2-NaCl) buffer, with a composition of 50 mM Tris-HCl, 5 mM MgCl_2_, 5 mM glucose, 100 mM KCl, and 200 mM NaCl at pH 7.5 (34). To simulate environmental conditions affecting motility, various reagents such as Ficoll as Newtonian fluid, methylcellulose as non-Newtonian fluid, bile acid, and mucin were introduced (all from Wako Pure Chemical, Osaka, Japan).

### Induction of *V. cholerae* into filamentous form

For filamentation, we used a solution containing 25 mg/mL yeast extract and 10 µg/mL ampicillin (Wako Pure Chemical) in artificial sea water (Sigma-Aldrich, MO, USA) (14). This solution was stored at 4°C to preserve its effectiveness. A single colony of *V. cholerae* was inoculated into 5 mL of fresh LB broth and incubated at 37°C with shaking until it reached an optical density at 600 nm (OD_600_) of 0.5–0.6. The cells were then centrifuged, resuspended in the induction solution, and incubated at 20°C in the dark without shaking. Morphological changes to filamentous form were monitored hourly using dark-field microscopy, with filamentous cells displaying intact motility typically observed after 3 hours of incubation.

### Electron microscopic observation

*V. cholerae* cells were prepared for transmission electron microscopy (TEM) by negative staining with 2% (wt/vol) phosphotungstic acid (Thermo Scientific, MA, USA), adjusted to pH 7.0 using NaOH. The staining process involved placing a drop of the bacterial suspension onto a carbon-coated copper grid and allowing it to adhere for 1–2 minutes. Excess liquid was then carefully removed with filter paper, and the grid was immediately stained by adding a drop of the phosphotungstic acid solution. After 1 minute, the excess stain was removed by blotting with filter paper, and the grid was air-dried. The stained samples were observed using a transmission electron microscope (JEM-1230R, JEOL, Japan) operating at an acceleration voltage of 120 kV. Images were captured to observe the morphology of both comma-shaped and filamentous *V. cholerae* cells.

### Observation of bacterial motility

Bacterial motility was observed using a dark-field microscope (Nikon Eclipse Ci-L Plus, Nikon, Tokyo, Japan) with one-sided illumination (35). Cell movement was recorded at 120 fps using a high-speed CMOS camera (Imaging Source, Taipei, ROC). Videos were analyzed quantitatively using ImageJ (NIH, MD, USA) and Excel (Microsoft, WA, USA), following established methodologies (19, 36, 37).

### Kinematic parameters of swimming *V. cholerae* cell

The geometric parameters of the *V. cholerae* cell bodies were measured and analyzed using ImageJ software. Modified resistive force theory and Stoke’s law were applied to estimate the swimming force for the comma-shaped and filamentous cells and the torque generated by the rotation of the filamentous cell body (38–41). The estimated swimming force was calculated based on the drag force acting on the cell body. The force required for swimming, denoted as $F_{swim}$, is approximated to be equal to the total drag force, $F_{drag}$, for straight-line swimming:


(1) $$F_{swim}=F_{drag}$$

The total drag force $F_{drag}$ along the comma-shaped cell body:


(2) $$F_{drag}=6\pi \eta rv$$

Equation (2) is the drag force on swimming comma-shaped cell, where η is the viscosity of the medium, r is the radius of the bacterium, and v is the swimming speed.

The total drag force $F_{drag}$ was derived by integrating different drag forces, dF, along the filamentous cell body:


(3) $$F_{drag}=\int_{0}^{L}\left(c_{∥}v_{∥}+c_{⊥}v_{⊥}\right)dl$$

(4) $$dF=\left(c_{∥}v_{∥}+c_{⊥}v_{⊥}\right)dl$$

Equation (3) is the drag force on the swimming filamentous cell. In this equation, $c_{∥}$ and $c_{⊥}$ represent the parallel and perpendicular drag coefficient for motion, respectively. The velocity components, $v_{∥}$ and $v_{⊥}$, represent the translational and rotational speeds of each segment of the helical cell body, here we used the measured values of the swimming speed and rotation rate, respectively. The drag coefficients and the velocity components were calculated from the geometric parameters of the cell and the viscosity of the medium:


(5) $$c_{∥}=\frac{4\pi \eta }{\ln\left(\frac{L}{r}\right)+\frac{1}{2}}$$

(6) $$c_{⊥}=\frac{8\pi \eta }{\ln\left(\frac{L}{r}\right)+\frac{1}{2}}$$

The length (L) and radius (r) of the cell body were all determined (Table S2). The viscosity of the medium (η) was measured using a tuning-fork viscometer (SV-10, A&D Company, Tokyo, Japan), as listed in Table S1.

The total torque exerted by the rotation of the cell was calculated by integrating the differential torque along the length of the filamentous cell body:


(7) $$\tau_{total}=\int_{0}^{L}d\tau =\int_{0}^{L}\left(c_{⊥}v_{⊥}\right)dl$$

### Reversal behavior during swimming

To observe the reversal behavior of filamentous *V. cholerae* during swimming, a suspension of these cells was introduced into a flow chamber (Fig. 4A) containing liquid bile and 5% mucin, creating a contiguous area of liquid and mucin with a distinct border between the two phases (42–44). This setup was previously developed by Abe et al. (42), allowing for the detailed observation of bacterial movement at the interface of the two phases. The movement of the bacterial cells at this border area was closely monitored using a dark-field microscope (Nikon Eclipse Ci-L Plus, 40×/0.75, Nikon, Tokyo, Japan). The observed movements were recorded using a CMOS camera at a frame rate of 90 fps, for further detailed analysis.

### Capillary assay

Comma-shaped and filamentous *V. cholerae* cells were adjusted to a concentration of 10^5^ cells/mL using a counting chamber (Funakoshi, Tokyo, Japan) (36). The bacterial cells were then suspended in 0.5 mL of liquid bile and transferred to a 1.5 mL microtube, serving as the bacterial suspension (Fig. 4E, upper). Glass capillaries containing 10 µL of 5% mucin were inserted into the bacterial suspension and incubated at room temperature. At time points of 0, 30, 60, 90, and 120 minutes, the capillary contents were collected, serially diluted, and plated on Luria-Bertani agar. The plates were incubated at 37°C for 18 hours, after which colony-forming units (CFUs) were counted to determine bacterial penetration into the mucin layer.

### Assessment of ATP synthesis

ATP levels in *V. cholerae* were measured using a thermostable luciferase-based ATP detection kit (BacTiter-Glo; Promega, Madison, WI, USA) following the manufacturer’s instructions. Briefly, *V. cholerae* cells from the induction solution were collected by centrifugation at 8,000 × *g* for 2 minutes and resuspended in 100 µL of phosphate-buffered saline (PBS) to a final concentration of 10^4^ cells/mL. Next, 100 µL of the luciferase reagent was added to each microtube, and the contents were mixed using an orbital shaker for 1 minute. Luminescence was measured using a GloMax 20/20 luminometer (Promega).

### CtxA gene expression measurement

Briefly, induction of filamentous *V. cholerae* was performed, followed by incubation at the specified time points, samples underwent centrifugation and were resuspended in 1 mL TRIzol (Invitrogen, MA, USA) for RNA extraction, utilizing the Qiagen RNeasy Kit for extraction and the TURBO DNA-free Kit (Ambion, TX, USA) for DNA digestion, in accordance with the manufacturers’ instructions. cDNA synthesis was conducted using the High Capacity cDNA Reverse Transcription Kit (Applied Biosystems, MA, USA), and qPCR analysis was carried out on two 384-well plates employing PowerUp SYBR Green Master Mix (Applied Biosystems) on QuantStudio 6 Flex Real-Time PCR system (Applied Biosystems). Primers used for analysis are as follows: groEL forward 5′-ATGATGTTGCCCACGCTAGA-3′, groEL reverse 5′-GGTTATCGCTGCGGTAGAAG-3′ (45), *ctxA* forward 5′-TTGGAGCATTCCCACAACCC-3′, and *ctxA* reverse 5′-GCTCCAGCAGCAGATGGTTA-3′ (46). Data analysis was executed using PRISM GraphPad v10.1.2, with statistical significance assessed *via* one-way ANOVA on relative expression levels (Livak Method). The housekeeping gene groEL served as a control in qPCR experiments.

### Direct fluorescent antibody assay

Watery stool samples were collected from cholera patients at the International Centre for Diarrhoeal Disease Research, Bangladesh (icddr,b), Dhaka, Bangladesh, and immediately used for the direct fluorescent antibody (DFA) assay. In the assay procedure, 1 mL of each concentrated sample was transferred to an Eppendorf tube and fixed with 110 µL of 37% formaldehyde. A direct fluorescent-monoclonal antibody staining kit (Cholera DFA, New Horizons Diagnostics Corporation, MD, USA) was utilized for the detection of filamentous *V. cholerae* cells (47). According to the manufacturer’s instruction, thin smears were prepared by applying 5 µL of each sample and control to separate wells, evenly spreading to cover the surfaces. After air drying at room temperature, these smears were fixed again with 5 µL of absolute ethanol and air-dried. For detection, 5 µL of either Cholera DFA reagent (for *V. cholerae* O1) or Bengal DFA reagent (for *V. cholerae* O139) was added to each well. The slides were then incubated in a dark, moist chamber at 37°C for 30 minutes, followed by thorough washing with PBS in a light-protected environment and subsequent air drying in the dark. A drop of fluorescent mounting medium was applied and covered with a cover slip. The final examination of the slides was conducted under a fluorescent microscope using an excitation wavelength of 490 nm and an emission wavelength of 520 nm.

### Statistical analysis

All experiments were performed at least three times. Statistical significance was analyzed using one-way analysis of variance (ANOVA) with Dunnett’s test and Mann-Whitney U test, as indicated in the figure legends (OriginPro 2023). *P < 0.05; **P < 0.01.

## Data Availability

The raw tracking data of this study have been deposited in Mendeley Data with the identifier: Xu, Jun (2024), “The Role of Morphological Adaptability in Vibrio cholerae's Motility”, Mendeley Data, V1, doi: 10.17632/vxzbjz2474.1. Source data are provided with this paper.

## SUPPLEMENTAL MATERIAL

Supplemental material mbio.02469-24-s0001.docx Figures S1 to S6; Tables S1 and S2; supplemental movie legends.

Movie S1 mbio.02469-24-s0002.mp4 Swimming filamentous *Vibrio cholerae* cells.

Movie S2 mbio.02469-24-s0003.mp4 Example of swimming filamentous cells under one-sided illumination dark-field microscopy.

Movie S3 mbio.02469-24-s0004.mp4 Movement of comma-shaped cells at the liquid-mucin border.

Movie S4 mbio.02469-24-s0005.mp4 Movement of a filamentous cell at the liquid-mucin border.

ASM does not own the copyrights to Supplemental Material that may be linked to, or accessed through, an article. The authors have granted ASM a non-exclusive, world-wide license to publish the Supplemental Material files. Please contact the corresponding author directly for reuse.
