# Supplementary material for: The role of morphological adaptability in Vibrio cholerae’s motility
Source: mBio. 2024 Nov 29;16(1):e02469-24. doi: 10.1128/mbio.02469-24 (PMC11708025; doi:10.1128/mbio.02469-24)
Supplement: Supplemental material — Figures S1 to S6; Tables S1 and S2; supplemental movie legends. [file mbio.02469-24-s0001.docx]

**Supplementary materials for**

The Role of Morphological Adaptability in *Vibrio cholerae*'s Motility

Jun Xu,^1^* Keigo Abe,^2^ Toshio Kodama,^3^ Marzia Sultana,^4^ Denise Chac,^5^ Susan M. Markiewicz,^5^ Hideyuki Matsunami,^6^ Erika Kuba,^1^ Shiyu Tsunoda,^1^ Munirul Alam,^4^ Ana A. Weil,^5^ Shuichi Nakamura,^2^ Tetsu Yamashiro^1^*

***Corresponding authors:** Jun Xu^a^*, Department of Bacteriology, Graduate School of Medicine, University of the Ryukyus, Nishihara, Okinawa, Japan. **Email:**  xujunbac@med.u-ryukyu.ac.jp

Tetsu Yamashiro^a^*, Department of Bacteriology, Graduate School of Medicine, University of the Ryukyus, Nishihara, Okinawa, Japan. **Email:**  tyamashi@med.u-ryukyu.ac.jp

**This supplementary materials file includes:**

Figures S1 to S6

Tables S1 to S2

Legends for Movies S1 to S4

**Other supporting materials for this manuscript include the following:**

Movies S1 to S4

Supporting Information Text

Figure S1.


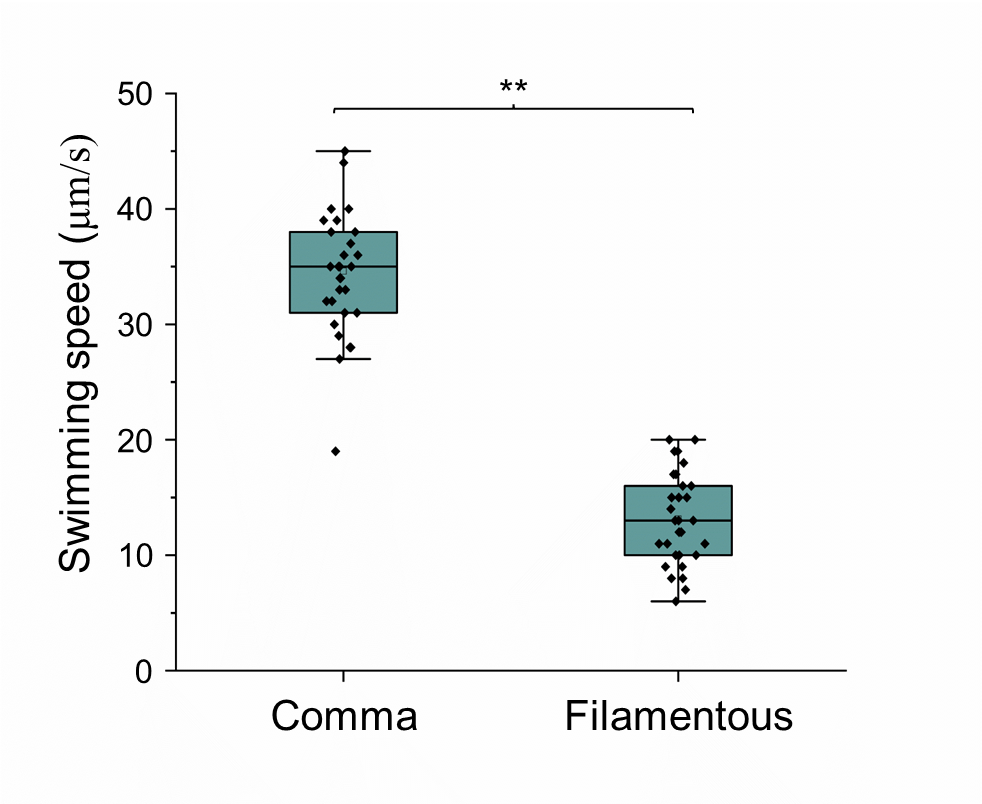


Fig. S1. The box charts show the swimming speed of two morphological forms of *V. cholerae* while swimming in LB media with bile. A one-way ANOVA with Dunnett’s test was conducted to assess the statistical significance of the observed differences in force exertion, with asterisks indicating significant differences (***P* < 0.01). The data is aggregated from a sample size of n=30 cells, compiled from three independent experimental trials.

Figure S2.


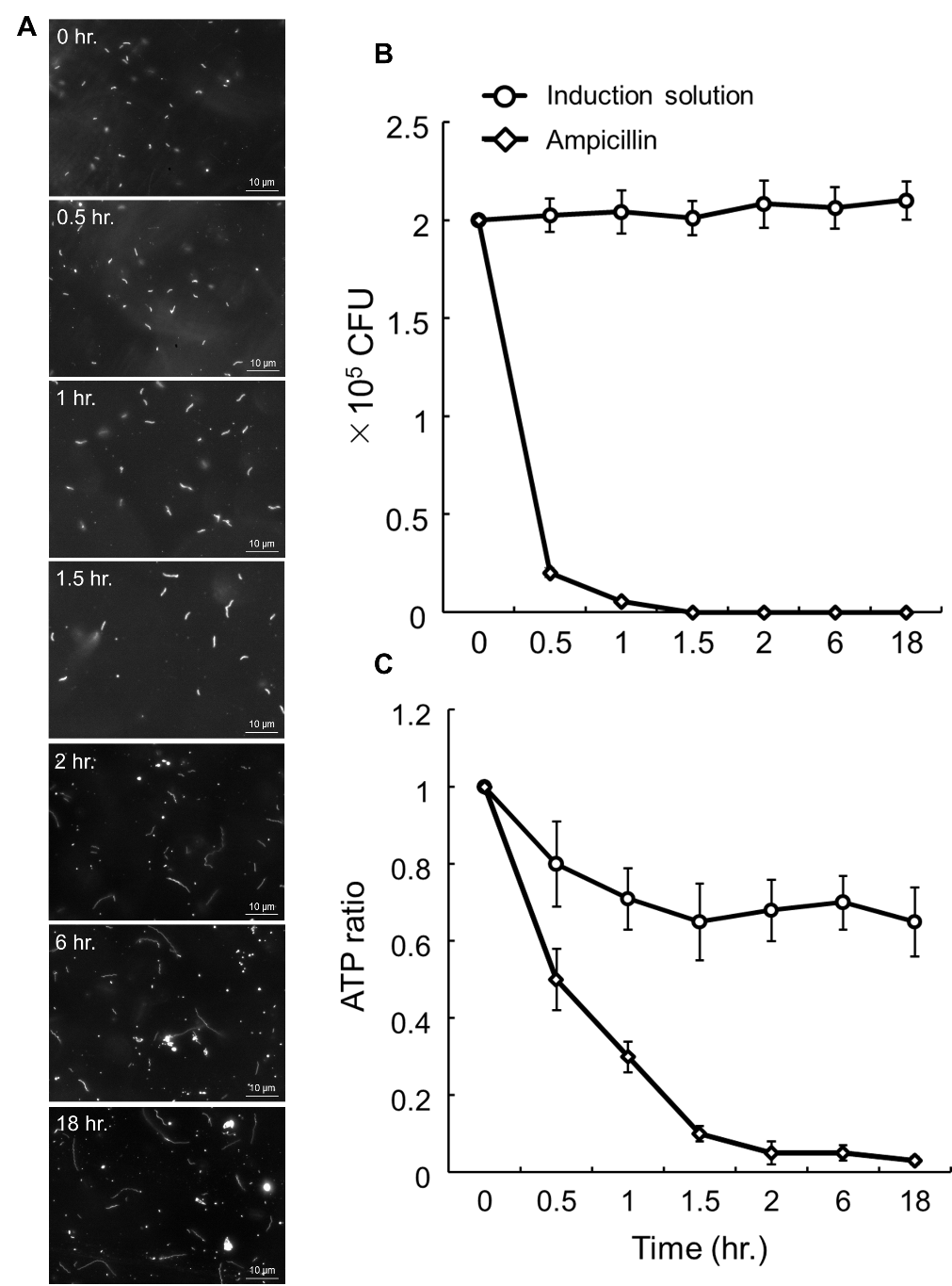


Fig. S2. Morphological and physiological changes in *V. cholerae* cells during filamentation. (A) Time-course images showing the morphological changes in *V. cholerae* cells over an 18-hour period following filamentation induction. (B) Cell count and (C) relative ATP ratio of *V. cholerae* cells in the induction solution, with the same solution containing 100 μg/mL ampicillin used as a control.

Figure S3.


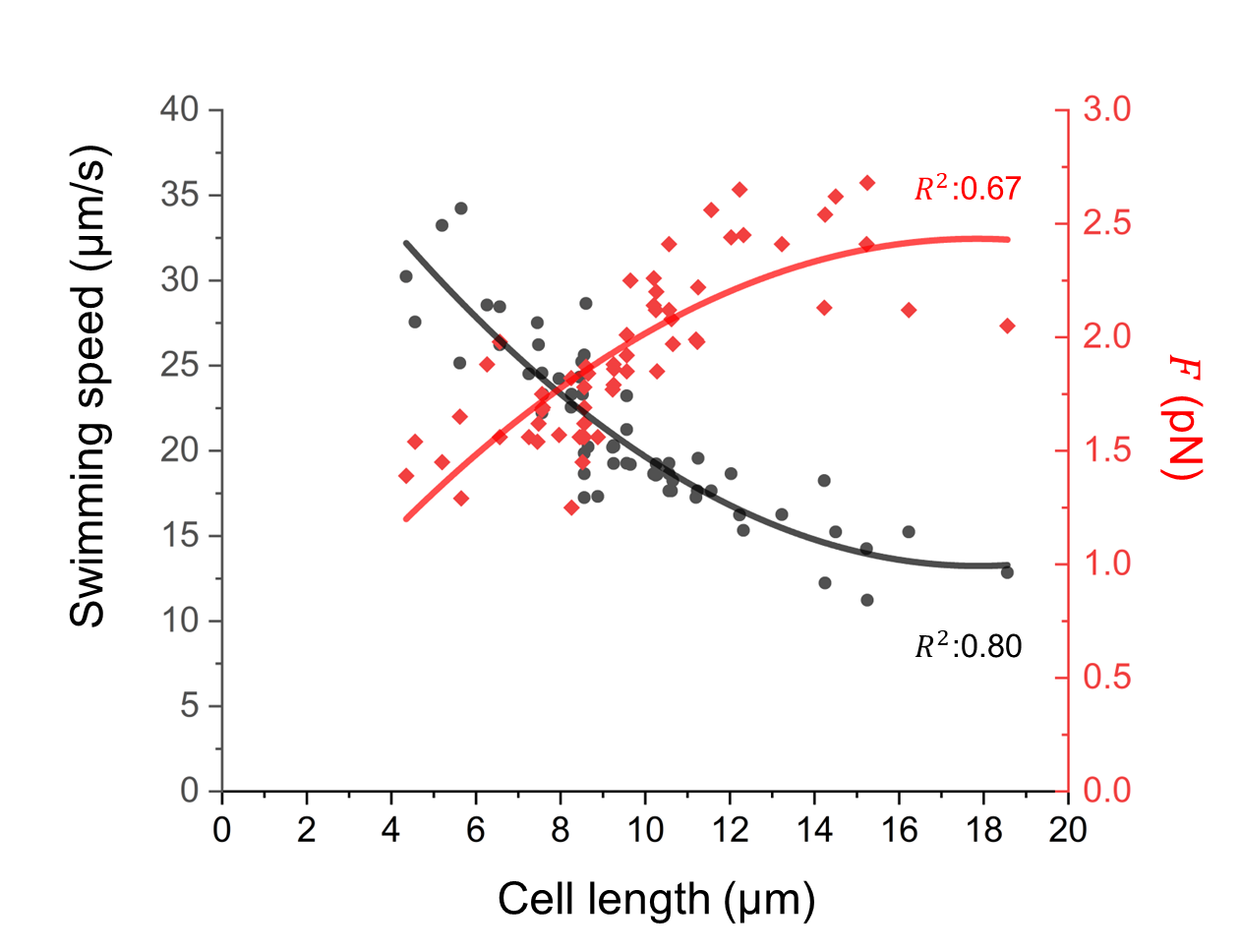


Fig. S3. Swimming speed and force of swimming filamentous *V. cholerae* cells correlated with cell length. Graph displays the relationship between cell length and two parameters: swimming speed (black dots) and the force exerted (red dots) on filamentous cells swimming in a medium with low viscosity. The black and red curves represent regression analyses for cell length-swimming speed and cell length-force (*F*), respectively.

Figure S4.


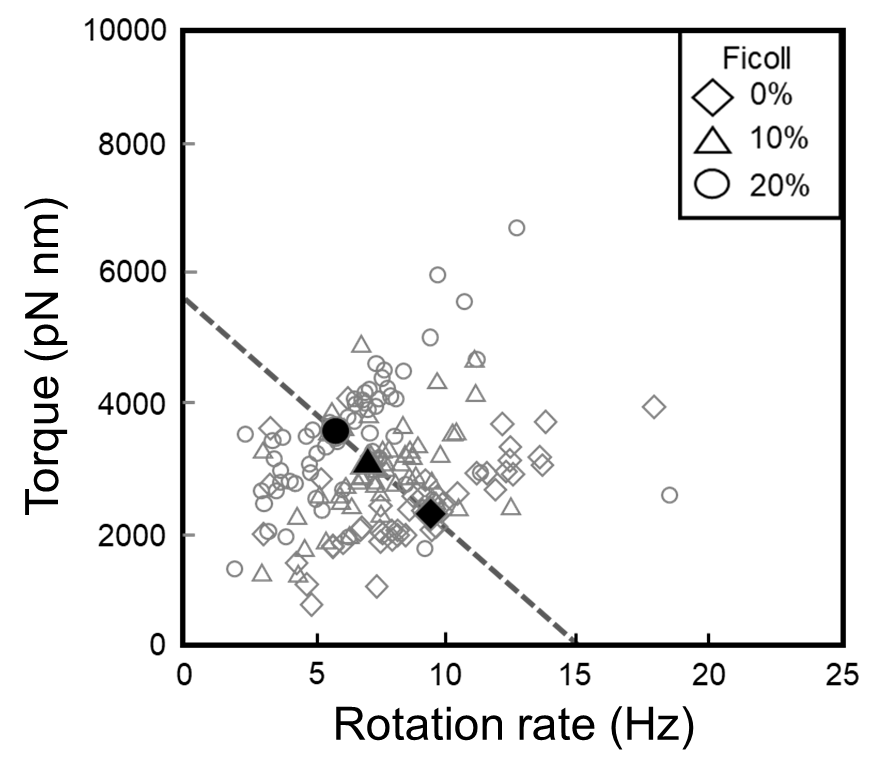


Fig. S4. Torque-speed relation of swimming filamentous *V. cholerae* cells. Graph shows the relationship between torque and rotation rate of the filamentous cell body in different viscosity conditions. Average values for each condition are presented in solid markers, with a fitted regression line (dashed line) illustrating the trend.

Figure S5.


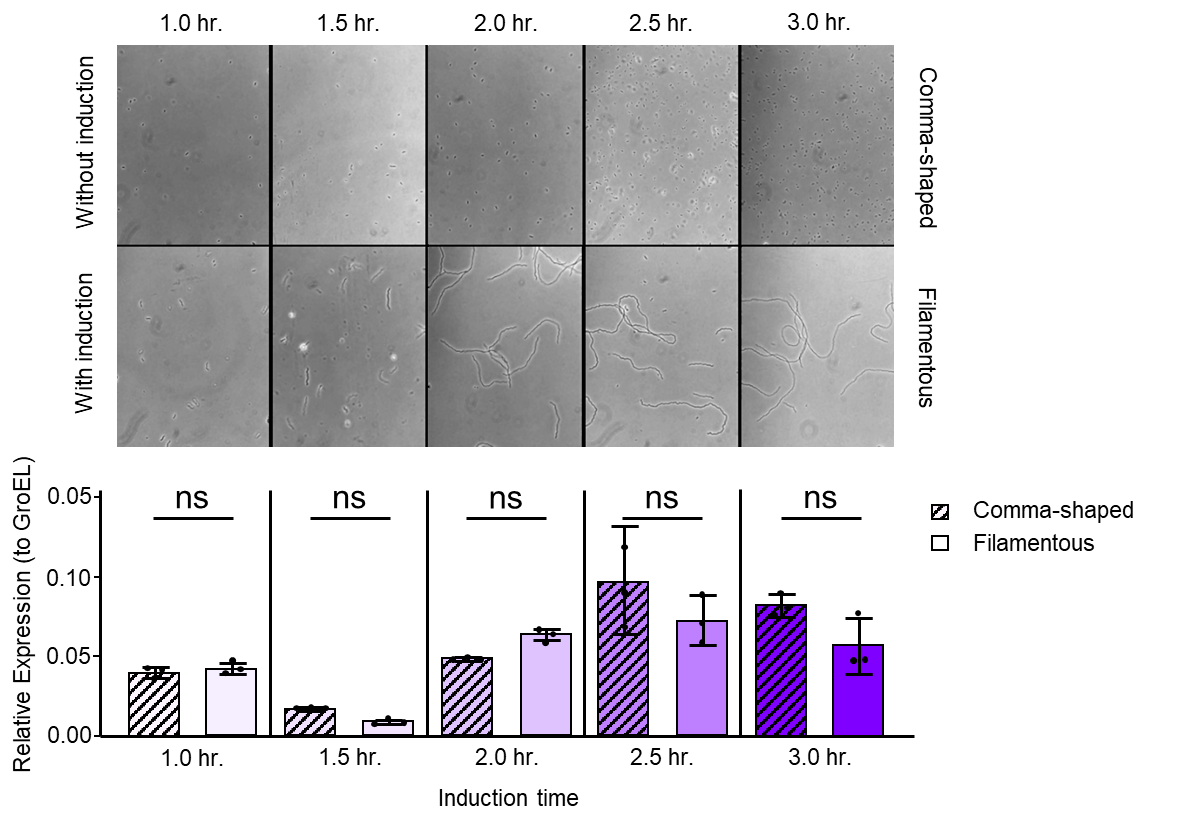


Fig. S5: Gene expression of *ctxA* in two morphologies of *V. cholerae*. This figure presents the time-course comparative analysis of *ctxA* gene expression between comma-shaped and filamentous *V. cholerae*. Morphologies were confirmed using phase microscopy on a microscope (Zeiss AxioStar Plus, Carl Zeiss, Oberkochen, Germany) with 400X magnification at each time point post-induction. Images (upper) are representative of the phenotypes observed during the assay. The qPCR results (lower) indicate no difference (ns) in the transcriptional levels of the *ctxA* gene between the comma-shaped and filamentous forms of *V. cholerae*.

Figure S6.


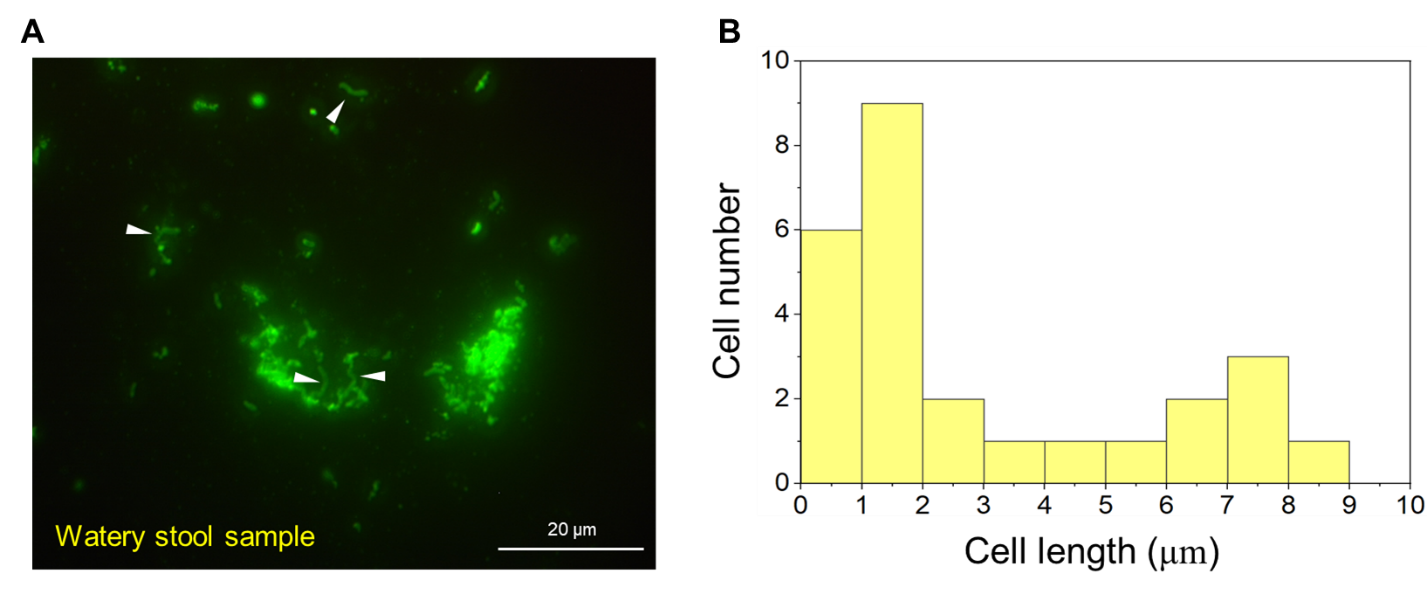


Fig. S6. Potential presence of filamentous *V. cholerae* in watery stool from patient with cholerae. (A) Epifluorescent microscopic image of a direct fluorescent antibody (DFA) assay conducted on watery stool samples from patient with cholera hospitalized at icddr,b Dhaka hospital. *V. cholerae* cells are labeled with a monoclonal antibody specific to *V. cholerae* O1, conjugated with fluorescein isothiocyanate (FITC), emitting green fluorescence. White arrows point to the filamentous cells, indicating their presence in clinical samples of cholera watery stool. (B) Histogram showing the cell length of *V. cholerae* cells presence in the watery stool sample.

Table S1.


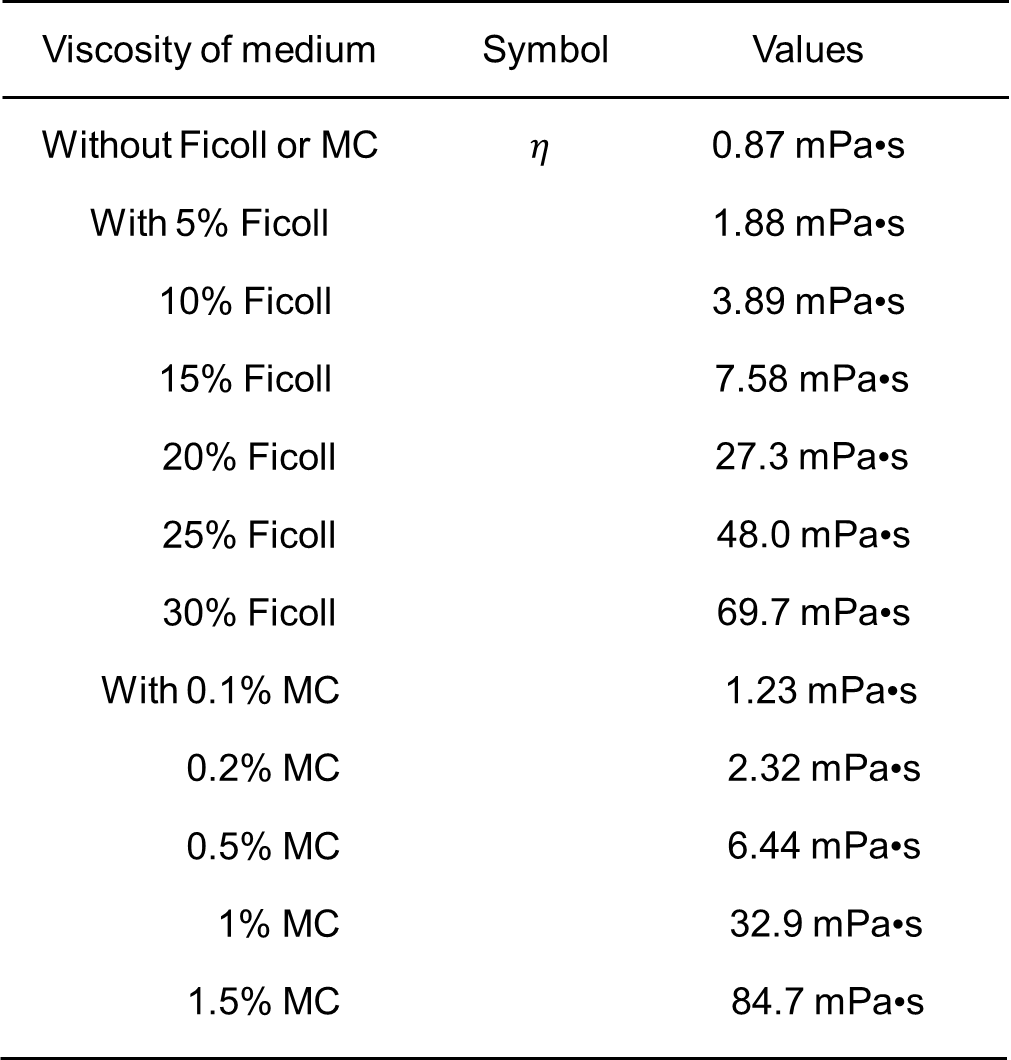


Table S1. Viscosity of the medium used in the study. MC=methylcellulose

Table S2.


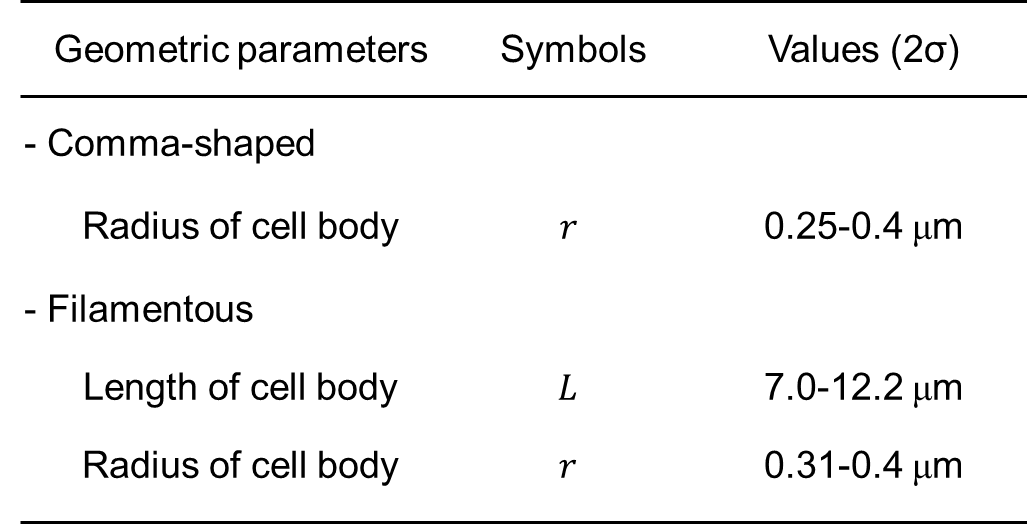


Table S2**:** Geometric parameters of filamentous *V. cholerae* cells. The geometric parameters were measured from a sample of 60 comma-shaped and 60 filamentous *V. cholerae* cells. Values falling within two standard deviations (2σ) of the mean are highlighted, representing the range that encompasses approximately 95% of all measured values, assuming a normal distribution. This approach provides a comprehensive overview of the typical geometric characteristics of *V. cholerae* cells under the study conditions.

**Movie S1 (separate file).** Swimming filamentous *Vibrio cholerae* cells.

Movie S2 (separate file). Example of swimming filamentous cells under one-sided illumination dark-field microscopy.

Movie S3 (separate file). Movement of comma-shaped cells at the liquid-mucin border.

Movie S4 (separate file). Movement of a filamentous cell at the liquid-mucin border.
